# Editorial: Polyamines in Plant Biotechnology, Food Nutrition, and Human Health

**DOI:** 10.3389/fpls.2020.00120

**Published:** 2020-02-19

**Authors:** Rubén Alcázar, Ana Margarida Fortes, Antonio F. Tiburcio

**Affiliations:** ^1^ Department of Biology, Healthcare and Environment, Section of Plant Physiology, Faculty of Pharmacy and Food Sciences, University of Barcelona, Barcelona, Spain; ^2^ Faculdade de Ciências de Lisboa, Department of Plant Biology, Biosystems and Integrative Sciences Institute, Universidade de Lisboa, Lisbon, Portugal

**Keywords:** polyamines, agriculture, climate change, health, nutrition, metabolism, plant protection, food

Polyamines are small polycations derived from arginine and/or ornithine. These compounds are present in all living organisms and play common and organism-specific functions. Polyamines are present in most food products of plant and animal origin, thus having an impact on human nutrition and health. In this Topic, we aimed to cover both basic and applied research on polyamines in the areas of plant biotechnology, food nutrition, and human health.

In plants, the most abundant polyamines are putrescine (Put), spermidine (Spd), and spermine (Spm). The control of polyamine levels is achieved through regulation of their biosynthesis, catabolism, and transport, which are modulated by the environment. Past and current research on polyamines has investigated basic processes of polyamine homeostasis, as a mean to obtain crops better adapted to climate change. The discovery of polyamine signaling pathways will also help in reaching this major goal. In this Topic, Zhao et al. report on the characterization of the Ornithine decarboxylase (ODC) enzyme from *Hyosciamus niger*, involved in Put biosynthesis. The ODC enzyme exhibits higher catalytic efficiency than other plant ODCs reported so far, thus providing an ideal candidate gene for polyamine biosynthesis engineering. Sekula and Dauter report on the crystal structure of Agmatine iminohydrolase involved in Put biosynthesis from arginine and identify that the dimeric assembly of monomers is drastically different from the bacterial enzyme. The same authors also obtained crystals of spermidine synthase, involved in Spd formation, and characterized the structures of the two dimeric enzyme isoforms in Arabidopsis (Sekula and Dauter).

Plants also contain thermospermine (tSpm), a less abundant but very relevant polyamine synthesized from Spd by thermospermine syntase (tSPM), in a reaction that is conserved throughout the plant kingdom (Solé-Gil et al.). According to these authors, tSPM might play developmental and/or stress-related roles in addition to the confirmed function in regulating xylem cells maturation, in both non-vascular and vascular plants. Ishitsuka et al. have shown that the response to tSpm is conserved in dicots and monocots and plays a role in translational enhancement that could be eventually applied as a biotechnological tool in animal and fungal systems. The work by Zarza et al. shows that Spm triggers a quick phosphatidic acid response in Arabidopsis, which is mainly mediated by phospholipase D. Their results suggest the participation of phosphatidic acid in Spm perception.

The cellular content of polyamines is also regulated by degradation mediated by diamine and polyamine oxidases (DAO and PAO). These catabolic processes and their diverse functions in plants have been reviewed by Wang et al. and include the involvement of polyamine catabolism in fruit ripening, senescence, and stress responses. Arabidopsis has four genes homologs of the human histone demethylase LSD1 (LDL1-3 and FLD) that bear a flavin amine oxidase domain, and act differently in the control of flowering time (Martignago et al.). This contribution evidences the role of different epigenetic mechanisms in the control of plant development and defense and their impact on agronomical traits.

The participation of polyamines in many aspects of plant development is well known. In this regard, Nambeesan et al. report that Spd impacts floral organ identity and fruit set in tomato involving GA metabolism and signaling. These authors also suggested that altered polyamine ratios may regulate floral developmental processes. A role for free polyamines in pollination of *Pyrus communis* flowers is also suggested (Mandrone et al.). Both tSPM synthase and DAO are required for zygotic embryogenesis and vascular development in Scots pine (Vuosku et al.). According to the authors, specific manipulation of polyamine gene expression might provide a way to enhance somatic embryo production in recalcitrant Scots pine lines with important biotechnological applications. Furthermore, the work from Sobieszczuk-Nowicka et al. contributes to a better understanding of the cellular and molecular mechanisms underlying senescence-related cell death.

Numerous studies have reported increased levels of polyamines under conditions of abiotic and biotic stresses. In fact, Spm has been proposed as a plant defense activator to both type of stresses (Seifi and Shelp). Under abiotic stress, Spm promotes abscisic acid (ABA) biosynthesis (Marco et al.) and activates ABA-mediated signaling pathways (Seifi and Shelp). Spm also modulates oxidative/antioxidant responses (Seo et al.) and promotes transcription of several defense-related genes including some involved in polyamine metabolism (Fortes et al.). In contrast to glycophytes [i.e., pepper; (Piñero et al.)], the levels of Spd/Spm are high in halophytes, thus polyamines have been proposed as useful indicators of plant salinity adaptation (Bueno and Cordovilla).

On the other hand, under biotic stress Spm promotes jasmonic acid-mediated signaling pathways (Seifi and Shelp), modulates oxidative/antioxidant responses (Seo et al.) and promotes transcription of several stress-related genes including PAOs (Menendez et al.). High Spm content in maize genotypes has been implicated in higher resistance to *Aspergillus flavus* and aflatoxin contamination (Majumdar et al.). So far, the role of Put during defense has remained elusive. In this Topic, Liu et al. report that this polyamine contributes to amplification of pathogen-associated molecular pattern (PAMP)-triggered immunity (PTI), through ROS production leading to enhanced disease resistance against bacterial pathogens. Menendez et al. suggest a link between polyamines and abiotic stress mitigation in legumes by symbiosis with fungi and bacteria. Furthermore, several authors (Bueno and Cordovilla; Fortes et al.,; Sobieszczuk-Nowicka et al.,; Vuosku et al.,; Wang et al.) highlight that manipulation of polyamine contents can be used to increase crop productivity and quality under more sustainable conditions by improving abiotic and biotic stress resilience. Investigations on the specific molecular mechanisms and signaling pathways by which polyamines exert these functions will follow in the near future.

Food is an important source of polyamines for human and animal nutrition. During the neonatal period, polyamine requirements are high. However, *de novo* biosynthesis decreases with age, which is the reason why polyamine dietary sources acquire a greater importance in aging populations. A wide range of polyamine concentrations can be found in all types of foods. The main polyamine in plant-based products is Spd, while Spm content is higher in animal-derived foods. Muñoz-Esparza et al. review the polyamine contents in breast milk and infant formula, as well as in different food of plant and animal origin. They also provide estimated levels of polyamine intake in different human populations. It is well known that dietary polyamines can affect human and animal health. Thus, exogenous polyamines (either dietary polyamines or produced by the gut microbiota) are able to induce longevity and cardioprotective effects in animals. Exogenous Spd or Spm improve glucose homeostasis and insulin sensitivity, and reduce hepatic fat accumulation in mouse models. Interestingly, increasing evidence indicate that polyamines act in the control of relevant human pathologies including cancer, immunological, neurological, and gastrointestinal diseases. Ramos-Molina et al. review how the diet influences circulating and local polyamine levels, and how the modulation of either polyamine dietary intake or endogenous production by gut microbiota can be used for potential therapeutic purposes.

Overall, this topic provides a good number of examples of basic and applied research on polyamines aiming at improving the quality of life of present and future human populations.

## Author Contributions

All authors listed have made substantial, direct, and intellectual contribution to the work and approved it for publication.

## Funding

Research by RA and AT are supported by the Agencia Estatal de Investigación (AEI) and the Fondo Europeo de Desarrollo Regional (FEDER) BFU2017-87742-R grant of the Programa Estatal de Fomento de la Investigación Científica y Técnica de Excelencia (Ministerio de Economía y Competitividad, Spain). Research by AF is supported by project PTDC/ASP-HOR/28485/2017 and UID/MULTI/04046/2019 Research Unit grant from FCT, Portugal (to BioISI).

## Conflict of Interest

The authors declare that the research was conducted in the absence of any commercial or financial relationships that could be construed as a potential conflict of interest.

